# Laparoscopic closure of the fascial defect combined with medial umbilical ligament reinforcement for incarcerated pediatric direct inguinal hernia: a case report with emergency management insights

**DOI:** 10.3389/fmed.2026.1840599

**Published:** 2026-04-30

**Authors:** Guanghua Zhang, Ming Sun, Jun Shu, Hongqiang Bian, Qin Guo, Jun Yang

**Affiliations:** 1 Department of General Surgery, Wuhan Children’s Hospital, Tongji Medical College, Huazhong University of Science and Technology, Wuhan, China; 2 Department of Ophthalmology, Wuhan Children’s Hospital, Tongji Medical College, Huazhong University of Science and Technology, Wuhan, China

**Keywords:** incarceration, laparoscopic repair, medial umbilical ligament, pediatric direct inguinal hernia, pediatric emergency surgery, point-of-care ultrasound

## Abstract

**Objective:**

Pediatric direct inguinal hernia (DIH) is an extremely rare congenital abdominal wall defect, accounting for less than 4% of all pediatric inguinal hernias. Its clinical manifestations overlap highly with indirect inguinal hernia (IIH), leading to frequent diagnostic dilemmas in emergency settings, especially for incarcerated cases. This single-case report aims to describe a case of incarcerated pediatric DIH and elaborate on the emergency diagnostic and therapeutic approach, to provide a detailed reference for managing similar cases.

**Methods:**

A 15-month-old male infant with left incarcerated DIH was admitted to the emergency department. Point-of-care ultrasound (POCUS) was performed to confirm the diagnosis by identifying the herniation pathway through Hesselbach’s triangle. Laparoscopic closure of the fascial defect combined with medial umbilical ligament reinforcement was implemented without synthetic mesh implantation, in line with the physiological characteristics of pediatric abdominal wall development.

**Results:**

The infant was accurately diagnosed via POCUS within 2 h of admission, and emergency laparoscopic surgery was completed within 6 h (including time for diagnosis, preoperative optimization, and mandatory fasting). The operation duration was 15 min with an estimated blood loss of 1 mL. Postoperative recovery was uneventful, and the infant was discharged on postoperative day 1. Follow-up at 1, 2, 3 and 6 months showed no hernia recurrence, with normal abdominal wall development.

**Conclusion:**

In this case, POCUS was instrumental in the emergency differential diagnosis. Laparoscopic defect closure combined with medial umbilical ligament reinforcement, which avoids synthetic mesh, appeared to be safe and feasible with good short-term outcomes. This report highlights a diagnostic and therapeutic pathway for a rare condition. As a single-case report, these findings are hypothesis-generating and require validation in larger studies. Future prospective studies with longer follow-up are needed to confirm the efficacy and generalizability of this approach.

## Introduction

Inguinal hernia is one of the most common congenital surgical conditions in children, with indirect inguinal hernia (IIH) accounting for more than 95% of all cases (1). In contrast, pediatric direct inguinal hernia (DIH) is extremely rare, with a reported intraoperative detection rate of 0.1–3%, resulting from congenital hypoplasia of the transversalis fascia within Hesselbach’s triangle (1, 2). Unlike adult DIH, which is mostly acquired due to chronic increased abdominal pressure, pediatric DIH is a developmental anomaly with no obvious gender predilection (3).

The high overlap in clinical manifestations between DIH and IIH poses a major diagnostic challenge in emergency settings, especially for incarcerated cases. Previous studies have reported misdiagnosis of pediatric DIH as incarcerated IIH, leading to inappropriate open surgery and subsequent recurrence (4). Although sporadic case reports of pediatric DIH exist in the literature, most focus on elective cases, with limited detailed descriptions of emergency diagnostic workflows and pediatric-specific laparoscopic repair techniques, particularly for incarcerated presentations (3, 5).

Herein, we present this single-case report of a 15-month-old infant with left incarcerated DIH, focusing on the emergency diagnostic process utilizing point-of-care ultrasound (POCUS) and the details of a mesh-free laparoscopic repair technique. The aim is to contribute to the limited body of evidence on managing this rare pediatric emergency.

## Case presentation

A 15-month-old male infant (weight 11 kg, height 78 cm) was admitted to the Emergency Department of Wuhan Children’s Hospital, with a 1-month history of recurrent left inguinal mass and persistent protrusion for 3 h (Table 1). The infant had no significant past medical or surgical history. The parents reported that the mass first appeared 1 month prior, manifesting during crying, standing, or straining with defecation, and initially resolving spontaneously when recumbent. In the 3 h before admission, the mass remained persistently prominent even when the infant was calm and recumbent, with no accompanying symptoms such as abdominal pain, vomiting, fever, or constipation.

**TABLE 1:** Timeline of clinical events.

Time point	Event
T = 0 h	Symptom onset (persistent left inguinal mass)
T + 3 h	Admission to Emergency Department
T + 3.5 h	POCUS examination performed
T + 5 h	Pre-operative optimization completed, fasting period begins
T + 9 h	Emergency laparoscopic surgery commenced
T + 9.25 h	Surgery completed
Post-op day 1	Discharged from hospital
1, 2, 3, 6 months	Follow-up visits with POCUS
Planned 12, 24 months	Long-term follow-up visits

### Vital signs and physical examination

On admission, vital signs were stable: heart rate 95 beats/min, blood pressure 100/65 mmHg, peripheral oxygen saturation (SpO_2_) 100% in room air, and body temperature 36.8°C. Physical examination revealed a calm, cooperative infant with a soft, non-distended abdomen, normal bowel sounds, and no tenderness or rebound tenderness. A 4.5 cm × 2.0 cm firm, non-tender, irreducible hemispherical mass was palpable 1 cm superior to the pubic tubercle and medial to the left deep inguinal ring; the cough impulse sign could not be assessed due to the infant’s age. The mass did not extend to the scrotum; bilateral testes were descended with normal size and texture, and the transillumination test of the mass was negative.

### Laboratory investigations

Routine laboratory tests were performed in the emergency department: white blood cell count 6.8 × 10?/L, neutrophil percentage 65%, hemoglobin 125 g/L, platelet count 230 × 10?/L (all in line with the reference range for 15-month-old infants, *Zhu Futang Practical Pediatrics* 8th Edition); serum electrolytes, liver function, renal function, coagulation function, and urine routine were all within age-appropriate pediatric reference ranges.

In this case, serum lactate was not measured. The clinical decision was based on the patient’s stable hemodynamic status throughout observation, the absence of clinical signs suggestive of advanced bowel ischemia (e.g., peritonitis, systemic toxicity), and the reassuring POCUS findings showing preserved blood flow. We acknowledge that incorporating serum lactate into the routine laboratory assessment for future similar cases could provide an additional objective parameter.

### Imaging examination and diagnosis

**FIGURE 1:**
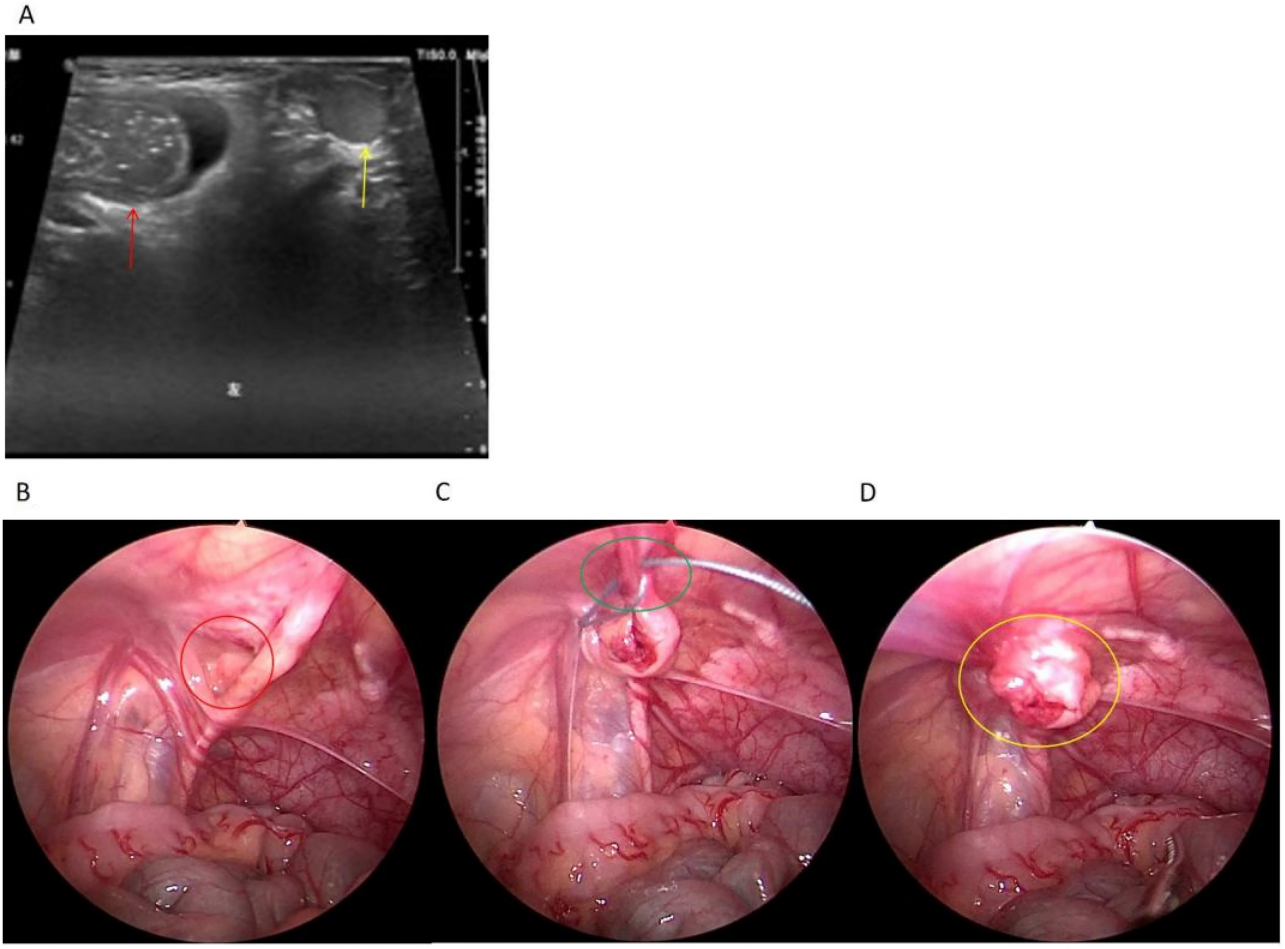
Combined POCUS and laparoscopic surgical images of left incarcerated pediatric DIH. **(A)** POCUS image of the left inguinal region, a herniated sac-like structure is visible in the left Hesselbach’s triangle with a wide-based hernial sac neck (red arrow), herniated contents did not pass through the left deep inguinal ring and had no communication with the scrotal cavity (yellow arrow); **(B)** laparoscopic exploration reveals a 2.5 cm diameter fascial defect in the left Hesselbach’s triangle (red circle); **(C)** medial umbilical ligament is retracted to cover 85% of the defect surface (green circle) and fixed with 3 stitches (the first shot) **(D)** post-surgery visual of the left Hesselbach’s triangle, no synthetic mesh was implanted (yellow circle), the fascial defect was closed completely.

Immediate POCUS of the inguinal region and scrotum was performed using a high-frequency linear transducer (10–15 MHz). POCUS findings: a herniated sac-like structure was identified in the left Hesselbach’s triangle (bounded laterally by the inferior epigastric vessels, medially by the lateral border of the rectus abdominis muscle, and inferiorly by the inguinal ligament), with a wide-based hernial sac neck (diameter ∼2.5 cm), the hernial sac contained a small segment of intestinal loop; herniated contents did not pass through the left deep inguinal ring and had no communication with the scrotal cavity—a key distinguishing feature of DIH from IIH (Figure 1A). Color Doppler flow imaging (CDFI) showed no abnormal blood flow in the herniated contents and no signs of intestinal wall ischemia.

The differential diagnoses considered included incarcerated indirect inguinal hernia, acute lymphadenitis, strangulated hydrocele, and testicular torsion. The absence of overlying skin erythema or warmth argued against lymphadenitis. The transillumination test was negative, and the mass was superior to the scrotum, making a hydrocele unlikely. The presence of a palpable mass distinct from the testis, combined with a positive cremasteric reflex, effectively ruled out testicular torsion.

Based on the combination of persistent irreducibility for 3 h, the absence of spontaneous reduction, and the POCUS findings confirming herniation of bowel content, a diagnosis of incarcerated DIH was made. Contrast-enhanced abdominal computed tomography (CT) and magnetic resonance imaging (MRI) were not performed to avoid unnecessary radiation exposure and prolonged diagnostic time.

### Pre-operative optimization and surgical intervention

The infant was managed as an urgent surgical case. To minimize the risk of pulmonary aspiration under general anesthesia, adherence to fasting guidelines (6 h for solids, 4 h for clear fluids) was prioritized, as he was hemodynamically stable without signs of strangulation or peritonitis. During this period, he was under continuous monitoring in the emergency observation unit. The timing of surgery was therefore determined by the completion of safe fasting duration. This approach reflects our institutional protocol for stable, incarcerated hernias without evidence of compromise. Pre-operative optimization was completed within 2 h of admission: the infant was fasted for 6 h and deprived of water for 4 h to meet general anesthesia requirements; abdominal skin preparation was performed; a joint evaluation by the pediatric surgery and emergency anesthesia teams excluded surgical and anesthetic contraindications. Written informed consent for surgery and anesthesia was obtained from the parents after full explanation of the condition.

Laparoscopic closure of the fascial defect combined with medial umbilical ligament reinforcing repair was performed in the emergency operating suite under general endotracheal anesthesia. The reinforcement was indicated for the 2.5 cm fascial defect to reduce the recurrence risk, and synthetic mesh was avoided in line with the guideline recommendation that autologous tissue repair is preferred for young children to prevent abdominal wall development impairment. The infant was placed in the supine position with slight abduction of the lower limbs. Repeat physical examination after satisfactory anesthesia found that the left inguinal mass had resolved with spontaneous reduction of the hernial contents.

Surgical procedure: 3.0 mm and 5.0 mm transverse incisions were made on the left and right sides of the umbilical ring, respectively. Carbon dioxide pneumoperitoneum was established at an intra-abdominal pressure of 6 mmHg (the safe threshold for infants under 2 years old); a 5 mm trocar was inserted for the laparoscope, and a 3 mm trocar as the operating port. Laparoscopic exploration revealed a 2.5 cm diameter fascial defect in the left Hesselbach’s triangle with a visible direct inguinal hernial sac and no obvious hernial contents (Figure 1B). Following reduction, the previously incarcerated segment of bowel was carefully inspected laparoscopically. It appeared pink, with normal peristalsis and no signs of ischemia, necrosis, or serosal injury, corroborating the preoperative POCUS findings. A 2 mm skin and subcutaneous incision was made at the body surface projection of the left deep inguinal ring (approximately the lowermost abdominal transverse crease). A hernia needle with 2-0 polypropylene non-absorbable suture (Johnson & Johnson, X559) was inserted into the extraperitoneal space through the incision; with the assistance of atraumatic vascular forceps (to avoid and protect adjacent blood vessels), an intracorporeal purse-string suture was placed and ligated at the fascial defect of the direct inguinal hernia, with the suture knot positioned subcutaneously to achieve tension-free closure. For reinforcement, the medial umbilical ligament was retracted to cover approximately 85% of the defect surface —a decision made intraoperatively to balance complete coverage with minimizing tension on the mobilized ligament—and fixed with 3 stitches of the same suture to the transversalis fascia, which provided secure fixation with minimal foreign material (Figure 1C). No synthetic mesh was implanted to avoid interference with abdominal wall development (Figure 1D). Exploration confirmed bilateral closed processus vaginalis and no fascial defect in the right Hesselbach’s triangle. After confirming no abdominal bleeding or intestinal injury, the pneumoperitoneum was released, the trocars were withdrawn, the wound edges were apposed, the 5.0 mm incision was closed with a subcuticular suture with 5-0 absorbable surgical suture, and the wound was dressed with medical tissue adhesive. Both testes were confirmed to be at the scrotal base. The operation was completed uneventfully with an estimated blood loss of ∼1 mL and a total operative time of 15 min.

### Post-operative course and follow-up

The infant was transferred to the pediatric gastrointestinal surgery ward for postoperative observation and nursing. Anesthesia recovery was uneventful with no adverse reactions such as nausea, vomiting, or respiratory depression. The infant was fasted for 6 h postoperatively, then tolerated a clear liquid diet with gradual transition to a normal diet and good oral intake. The infant was able to ambulate independently 12 h postoperatively with no obvious surgical incision pain, no crying, and normal micturition and defecation. No postoperative complications occurred.

Follow-up at 1 week postoperatively showed primary healing of the surgical incision with no scar hyperplasia, redness, exudation, or hematoma formation. The infant was discharged on postoperative day 1 with discharge instructions: avoid strenuous activities (e.g., running, jumping) for 1 month; prevent severe cough, excessive crying, and constipation to reduce abdominal pressure; resume normal daily activities after 1 month. Abdominal POCUS follow-up at 1, 2, 3 and 6 months postoperatively showed the fascia thickness of the left Hesselbach’s triangle was 3 mm (consistent with the healthy right side), no hernia recurrence, normal abdominal wall development, and growth parameters matching the 15–21 month infant growth curve. Long-term follow-up with abdominal ultrasound at 12 and 24 months postoperatively is planned to further evaluate the long-term prognosis.

## Discussion

Pediatric direct inguinal hernia (DIH) is an extremely rare condition. This report describes the emergency management of an incarcerated DIH in a 15-month-old infant.

### Diagnostic challenges and the value of POCUS in this case

The main diagnostic challenge lies in differentiating DIH from the much more common indirect inguinal hernia (IIH), as their clinical presentations are highly similar (3, 6). In the emergency setting, this is compounded by the patient’s age. The key anatomical difference—the herniation pathway relative to the inferior epigastric vessels—is difficult to ascertain by physical exam alone.

In this emergency, point-of-care ultrasound (POCUS) served as the decisive diagnostic tool. It rapidly and non-invasively identified the hernial sac medial to the inferior epigastric vessels within Hesselbach’s triangle, without extension to the scrotum, confirming the diagnosis of DIH and excluding IIH and testicular torsion (1, 6, 7). This allowed for prompt surgical planning. The utility of POCUS for diagnosing pediatric inguinal hernias and for differentiating subtypes is supported by recent literature (8–10).

The preoperative identification of a direct hernia via POCUS has specific implications for surgical strategy beyond confirming the diagnosis. First, it informs the surgeon that the defect is located in the inherently weaker Hesselbach’s triangle, reinforcing the need for a durable repair that addresses the fascial deficiency. Second, it prompts preoperative planning for a potential reinforcement procedure using autologous tissue, as synthetic mesh is contraindicated in young children. This foreknowledge allows for optimal patient positioning, port placement, and preparation of necessary sutures, thereby streamlining the emergency operative procedure.

### Surgical treatment and pediatric-specific principles

Laparoscopic surgery is well-suited for this condition, offering excellent visualization, minimal invasiveness, and the ability to inspect the contralateral side (4, 5). In this case, it enabled a quick and precise repair. The total time from admission to surgery completion (6.25 h) included necessary diagnostic evaluation and adherence to safe fasting guidelines. While every effort is made to expedite care for incarcerated hernias, this timeline was deemed acceptable as the child remained stable.

The most critical principle in pediatric DIH repair is the avoidance of synthetic mesh, in contrast to adult practice (4, 11). To avoid potential interference with abdominal wall growth, we utilized an autologous tissue reinforcement technique. In this case, secure closure of the fascial defect was combined with reinforcement using the medial umbilical ligament—an autologous, tough structure in close anatomical proximity. This mesh-free approach adheres to pediatric-specific surgical principles. While the term ‘high ligation’ is conventionally used for indirect hernias, in this direct hernia repair, the analogous critical step was the secure, tension-free purse-string closure of the fascial defect in Hesselbach’s triangle. The technical details, such as the extent of coverage and number of sutures, were determined intraoperatively based on the defect size and tissue mobility.

### Insights from this case: emergency diagnostic pathway and technical nuances

This case offers two main insights for managing rare incarcerated pediatric DIH. First, it demonstrates a practical emergency pathway: integrating POCUS as a first-line tool to perform an anatomy-based, rapid differentiation between DIH and IIH, which is critical for correct surgical planning. Second, it provides a detailed example of a mesh-free, pediatric-appropriate laparoscopic technique. The use of the medial umbilical ligament for reinforcement represents a logical application of available autologous tissue. The use of adjacent autologous tissue for reinforcement in pediatric hernia repair is not novel in principle (11). The contribution of this report lies in the detailed description of its application in the specific context of an incarcerated pediatric direct inguinal hernia, along with the rationale provided for intraoperative technical decisions such as the extent of coverage and suture number, which are seldom elaborated in the literature.

### Limitations

As a single-case report, our findings should be interpreted with caution. The follow-up period is currently 6 months (mid-term), and while no recurrence was observed, long-term follow-up (e.g., 2–5 years) is planned and necessary to fully assess outcomes. Furthermore, the diagnostic accuracy of the POCUS features described cannot be calculated from a single case. Finally, the patient was a generally healthy infant, and outcomes may differ in neonates or children with comorbidities.

## Data Availability

The original contributions presented in the study are included in the article/supplementary material, further inquiries can be directed to the corresponding author.
